# Impact of bariatric surgery on oral anticancer drugs: an analysis of real-world data

**DOI:** 10.1007/s00280-024-04640-0

**Published:** 2024-03-01

**Authors:** Cedric Lau, Ma Ida Mohmaed Ali, Lishi Lin, Dorieke E. M. van Balen, Bart A. W. Jacobs, Bastiaan Nuijen, Robert M. Smeenk, Neeltje Steeghs, Alwin D. R. Huitema

**Affiliations:** 1 Department of Pharmacy and Pharmacology, The Netherlands Cancer Institute, Plesmanlaan 121, 1066 CX Amsterdam, The Netherlands; 2 Division of Pharmacology, The Netherlands Cancer Institute, Plesmanlaan 121, 1066 CX Amsterdam, The Netherlands; 3 Division of Medical Oncology, The Netherlands Cancer Institute, Plesmanlaan 121, 1066 CX Amsterdam, The Netherlands; 4 Department of Clinical Pharmacy, Albert Schweitzer Hospital, Albert Schweitzerplaats 25, 3318 AT Dordrecht, The Netherlands; 5 Department of Surgery, Albert Schweitzer Hospital, Albert Schweitzerplaats 25, 3318 AT Dordrecht, The Netherlands; 6 Department of Pharmacology, Princess Máxima Center for Pediatric Oncology, Heidelberglaan 25, 3584 CS Utrecht, The Netherlands; 7 Department of Clinical Pharmacy, University Medical Center Utrecht, Utrecht University, Heidelberglaan 100, 3584 CX Utrecht, The Netherlands

**Keywords:** Bariatric surgery, Oncology, Clinical decision support, Dose optimization, Therapeutic drug monitoring

## Abstract

**Purpose:**

The number of patients with bariatric surgery who receive oral anticancer drugs is rising. Bariatric surgery may affect the absorption of oral anticancer drugs. Strikingly, no specific drug dosing recommendations are available. We aim to provide practical recommendations on the application of oral anticancer drugs in patients who underwent bariatric surgery.

**Methods:**

Patients with any kind of bariatric surgery were extracted retrospectively in a comprehensive cancer center. In addition, a flowchart was proposed to assess the risk of inadequate exposure to oral anticancer drugs in patients who underwent bariatric surgery. Subsequently, the flowchart was evaluated retrospectively using routine Therapeutic drug monitoring (TDM) samples.

**Results:**

In our analysis, 571 cancer patients (0.4% of 140.000 treated or referred patients) had previous bariatric surgery. Of these patients, 78 unique patients received 152 oral anticancer drugs equaling an overall number of 30 unique drugs. The 30 different prescribed oral anticancer drugs were categorized as low risk (13%), medium risk (67%), and high risk (20%) of underdosing. TDM plasma samples of 25 patients (82 samples) were available, of which 21 samples post-bariatric surgery (25%) were below the target value.

**Conclusions:**

The proposed flowchart can support optimizing the treatment with orally administered anticancer drugs in patients who underwent bariatric surgery. We recommend performing TDM in drugs that belong to BCS classes II, III, or IV. If more risk factors are present in BCS classes II or IV, a priori switches to other drugs may be advised. In specific cases, higher dosages can be provided from the start (e.g., tamoxifen).

**Supplementary Information:**

The online version contains supplementary material available at 10.1007/s00280-024-04640-0.

## Introduction

The prevalence of obesity has increased worldwide. For patients with severe obesity and comorbidities, bariatric surgery is currently the most effective treatment [1]. The two most frequently performed bariatric surgical procedures are *sleeve gastrectomy* or *gastric sleeve* (SG) and *Roux-en-Y gastric bypass* (RYGB). SG involves excising about 75% of the stomach. RYGB involves creating a small gastric pouch of around 30 mL which is anastomosed to the distal limb of a jejunotomy performed at the mid-jejunum. Previously, older techniques such as *gastric banding* (GB) or *biliopancreatic diversion* (BPD)/*duodenal switch* have also been performed. Recently, other techniques such as *one-anastomosis gastric bypass* (OAGB) have been applied. OAGB is a type of gastric bypass with a longer gastric pouch and a single anastomosis between the gastric pouch and the jejunum.

All bariatric surgical procedures induce weight loss as a result of gastrointestinal changes in anatomy and physiology, influencing the pH and gastric motility [2]. As a result, the pharmacokinetics of orally administered drugs can be influenced due to altered absorption, distribution, metabolism and elimination. This has been shown for example for lithium [3], morphine [4] and anti-epileptics [5]. Guidelines have been developed for the use of several drugs in bariatric patients. For example, NSAIDs are discouraged after bariatric surgery due to the risk of minimal ulcers and gastrointestinal bleedings [6]. Furthermore, general guidelines recommend monitoring plasma drug levels more frequently for drugs requiring periodic plasma level control after bariatric surgery [6]. However, there are no specific drug dosing recommendations available for bariatric patients using anticancer drugs in any guidelines yet.

With increasing numbers of patients undergoing bariatric surgery worldwide [7], the number of cancer patients who underwent bariatric surgery is expected to increase too. Patients who underwent bariatric surgery gain a longer life expectancy than obese patients without bariatric surgery [8]. Orally administered anticancer drugs often possess an exposure-efficacy and/or an exposure-toxicity relationship. Therefore, underdosing or overdosing of these drugs has serious consequences, leading to either treatment failure or an increased risk of adverse events. Yet, this is a highly understudied area in patients who underwent bariatric surgery. In the literature, there are only several case reports on the use of tyrosine kinase inhibitors [9] and tamoxifen [10] in patients who previously underwent bariatric surgery. In these case reports, reduced plasma or serum levels of these drugs were observed, highlighting the suboptimal treatment of these patients using anticancer drugs.

The extent to which the absorption of an oral anticancer drug is affected by bariatric surgery depends on its physical and chemical properties. These include gastric acid-dependent solubility and the classification of a drug within the *Biopharmaceutics Classification System* (BCS) [11]. In short, the BCS class system is used to categorize a drug based on its solubility and permeability. A drug is highly soluble when the highest dose is soluble in 250 mL or less of aqueous media over the pH range of 1.2–6.8 at 37 ± 1 °C and high permeable when the absolute bioavailability is ≥ 85% [12]. Due to the unfavorable physical and chemical properties, several anticancer drugs have an enabling formulation, i.e., formulations making drugs more soluble and thus better absorbed [13].

To optimize treatments for future cancer patients, it is important to investigate the prevalence of bariatric patients using anticancer drugs and how this surgical intervention may affect the effectiveness and tolerability of oral anticancer drugs. Hence, we studied the prevalence of bariatric patients and the potential impact of bariatric surgery on the exposure to oral anticancer drugs in a large comprehensive cancer center. Based on this analysis, we aimed to propose practical recommendations for patients who underwent bariatric surgery receiving oral anticancer drugs.

## Methods

### Patients

This retrospective observational cohort study was conducted at the Netherlands Cancer Institute (NKI), Amsterdam, the Netherlands. Patients were included in this study when they were treated or referred to our hospital and had any kind of bariatric surgery in their medical history. The conduct of this observational study was approved by the Investigational Review Board of the NKI (IRBd23-007).

Data on patient characteristics, tumor characteristics, and medical treatments until 2023 April 6th were extracted from the electronic health record (EHR) HiX (Chipsoft, Amsterdam, the Netherlands). See Appendix 1 for used search terms. In the oral anticancer drug analysis, only oral anticancer drugs (ATC L01 or L02) were selected. Progestin was not regarded as an anticancer drug and was thus excluded from the analysis.

### Flowchart

**Fig. 1:**
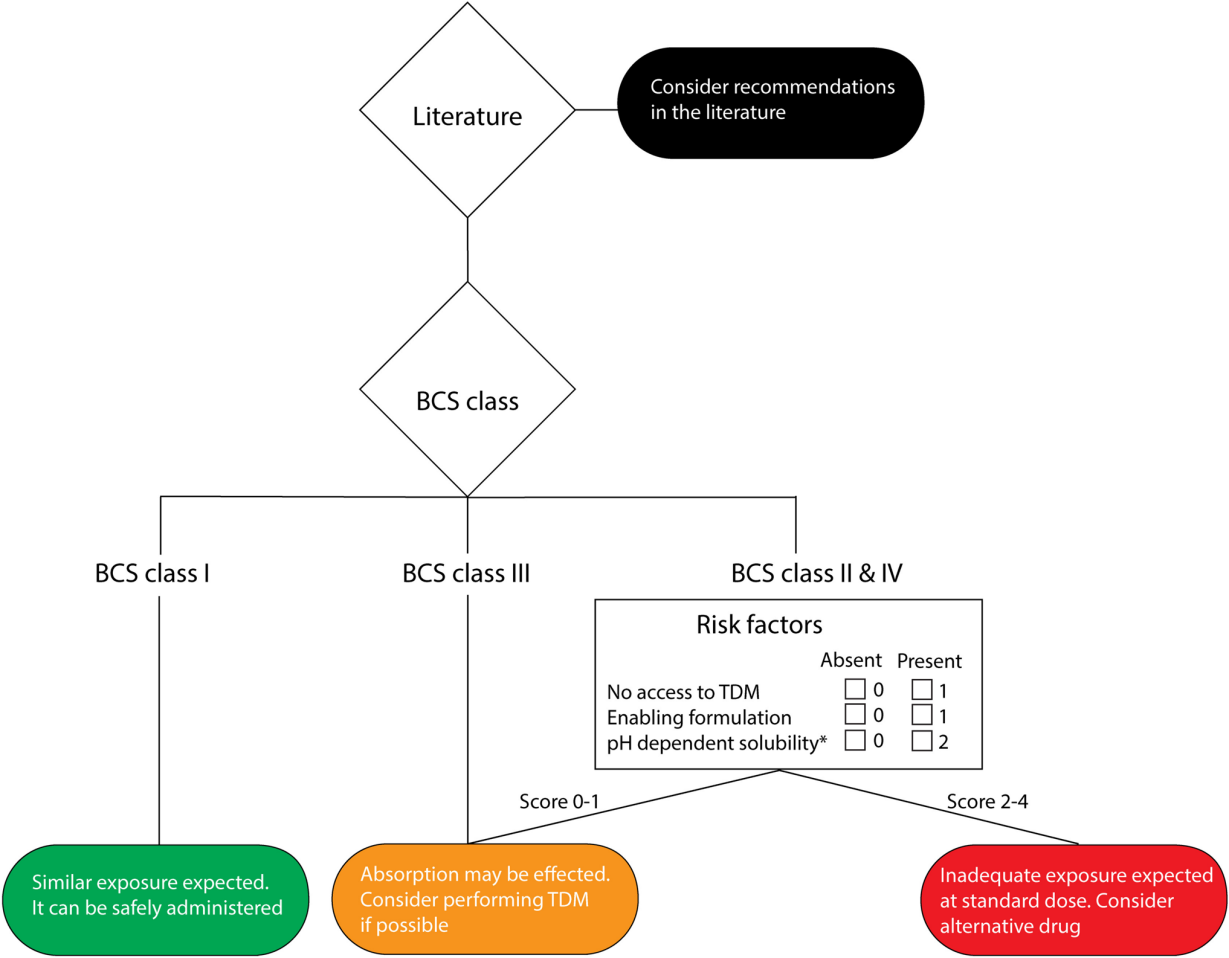
Flowchart for the use of oral anticancer drugs in patients who underwent bariatric surgery. The colors represent the risk of lower plasma samples in blood (green = low risk, orange = medium risk, red = high risk). Abbreviations: *TDM* Therapeutic drug monitoring. * If patients underwent SG longer than 2 years ago, their risk score for this item was set to 1 point

The expert opinion-based flowchart aimed to categorize the oral anticancer drugs for the risk of underdosing due to bariatric surgery. The drugs were categorized into low, medium, or high risk based on their BCS class (Fig. 1), which was based on information in recent reviews [14, 15]. For simplicity and clarity, this flowchart was proposed for patients who underwent SG, RYGB, or OAGB. Patients with GB were not incorporated, because GB was expected to have a limited effect on the absorption of oral anticancer drugs. GB only changes the size of the stomach opening and the volume of the functional part of the stomach. Subsequently, the flowchart was evaluated retrospectively with routine plasma samples from the NKI.

In the flowchart, BCS class I drugs (high solubility and permeability) were considered to be of low risk (green), as the changes in the gastrointestinal tract following bariatric surgery are not expected to negatively influence the absorption of these drugs. For BCS class II (low solubility and high permeability), III (high solubility and low permeability), and IV drugs (low solubility and permeability), changes in absorption were anticipated to be relevant and were classified as medium risk (orange) or high risk (red) when additional risk factors were present. These include drugs with pH-dependent solubility, drugs with an enabling formulation [13] and drugs with no possibility of performing TDM-guided dosing. For medium-risk drugs, we propose additional monitoring. This can be done by performing therapeutic drug monitoring (TDM) to prevent underdosing. On the other hand, for high-risk drugs, an excessive risk of inadequate exposure was expected. Therefore, we proposed a switch of therapy for this group of drugs.

Drugs with pH-dependent solubility were included as an additional risk factor, as bariatric patients have a higher gastric pH. However, the pH in the gastric pouch in SG patients returns to preoperative values after one year [16] and for the pH in RYGB patients remains high no matter the time after surgery [17]. So, we expected that over time SG patients become less prone to reduced absorption for drugs with pH-dependent solubility. This is why patients who underwent SG longer than two years ago may have a lower risk at decreased absorption than RYGB or OAGB patients. Drugs with an enabling formulation were also included as an additional risk factor, due to less time and less intestinal surface to be disintegrated and dissolved.

### Plasma samples and statistical analyses

Plasma samples of different oral anticancer drugs were collected as a part of routine clinical care. Plasma samples drawn at the time up to 9 months before or any time after the bariatric surgery, were included in the analyses to compare these plasma samples with previously published thresholds based on exposure–response and/or exposure-toxicity relationships [18, 19]. Plasma samples were excluded if they were drawn after therapy discontinuation or during an off-treatment period. Trough levels were estimated in clinical care by means of log-linear extrapolation [20] or specific population pharmacokinetic models. All statistical analyses were performed in R version 4.0.3 (R Foundation for Statistical Computing, Vienna, Austria).

## Results

Over eleven years, 571 patients were identified with any kind of bariatric surgery in their medical history. This was approximately 0.4% of the total number of patients treated or referred to our hospital in that period (140.000). The patient characteristics are shown in Table 1. In total, 80% of the patients were female and 51.5% underwent a RYGB. The most common type of bariatric surgery in the period between 1982–2004 was a GB. From 2004 onwards, the number of patients undergone RYGB and SG started to increase. The most diagnosed cancer type was breast cancer (28%).

**Table 1:** Patient characteristics

Patient characteristics	Patients (*n* = 571)
**Age at first cancer diagnosis (year)**	53.2 (15.6–80.1)
**Sex: female**	458 (80%)
**Bariatric surgery:**	
• RYGB	• 294 (51.5%)
• GB	• 83 (14.5%)
• SG	• 30 (5.3%)
• OAGB	• 6 (1.1%)
• Not specified	• 158 (27.7%)
**Type of cancer:**	
• Breast cancer	• 158 (27.7%)
• Sarcoma	• 30 (5.3%)
• Lung cancer	• 26 (4.6%)
• Melanoma	• 25 (4.4%)
• Colon carcinoma	• 25 (4.4%)
• Prostate cancer	• 20 (3.4%)
• Other	• 283 (50.3%)

### Oral anticancer drugs

Of the 571 patients with a type of bariatric surgery in the history, 152 unique patient-drug combinations were identified in the prescription data (Supplementary Table S1). In total, 78 unique patients were treated with one or more oral anticancer drugs (range 1–7). Most patients were prescribed tamoxifen (n = 42), followed by letrozole (n = 25), capecitabine (n = 17), and anastrozole (n = 13). In total, 30 different oral anticancer drugs were used and 20 of these drugs were categorized as medium risk (67%), six as high risk (20%), and four as low risk (13%) based on the flowchart in Fig. 1.

### Therapeutic Drug Monitoring (TDM) data

Routine TDM data were available from 2014 onwards. In total, 82 TDM samples were available from this patient population. Overall, 25 unique combinations of patient-plasma or serum level were identified. The data of three GB patients are shown separately in Supplementary Table S2, whereas the data of the remaining patients are shown in Table 2. A median of two plasma/serum samples per patient was available (range 1–13). The drugs for which TDM was performed most was endoxifen (n = 9), followed by palbociclib (n = 3). Of all TDM samples, 21 samples post-bariatric surgery (25%) in 11 patients fell below target.

**Table 2:** Overview of measured serum or plasma levels and used drug doses in patients before or after bariatric surgery

Drug (n patients) Risk based on flowchart	Type of surgery	Dosage	Time after surgery	Trough concentration^#^	Target concentra-tion^##^	Expected concentration range in non-bariatric patients	Expected trend in bariatric patients*	Previously reported trough concentrations in bariatric patients	Practical recommendation
Tamoxifen (n = 8) Medium risk	RYGB	20 mg OD	Before surgery (−8 m)	4.3 ng/mL	> 5.9 ng/mL	0–73.7 ng/mL (IQR 4.2—26.2 ng/mL) [30]	↓	Endoxifen concentrations not reported Tamoxifen concentrations: 14–52 ng/mL (ref: 77–274 for 10–30 mg OD) [10]	TDM-guided dosing
		30 mg OD	Before surgery (−1 m)	8.8 ng/mL					
	RYGB	20 mg OD	1 m	2.5 ng/mL					
		40 mg OD	4 m	3.8 ng/mL					
		20 mg OD	1 y	1.1 ng/mL					
	RYGB	20 mg OD	22 m	2.6 ng/mL					
		40 mg OD	3 y	6.4–7.3 ng/mL (2)					
		20 mg OD	3 y	3.1 ng/mL					
	RYGB	20 mg OD	Before surgery (−1 m)	13.7 ng/mL					
			6 m	8.1 ng/mL					
	SG	20 mg OD	7 y	2.7 ng/mL					
		40 mg OD	8 y	7.2 ng/mL					
	Unknown	20 mg OD	Before surgery (−1 m)	10.9 ng/mL					
	Unknown	20 mg OD	Before surgery (−4 m)	2.35 ng/mL					
		30 mg OD	Before surgery (−1 m)	3.47 ng/mL					
		40 mg OD	2 m	5.8 ng/mL					
	Unknown	40 mg OD	3 y	10.9 ng/mL					
Pazopanib (n = 2) High risk	RYGB	800 mg OD	5 y	10–15 mg/L (2)	> 20 mg/L	800 mg OD: 29.7 mg/L (range 0.0—83.2) [31]	↓	[25]	Consider alternative drug. *Alternatively, TDM-guided dosing*
		400 mg BD	5 y	13–23 mg/L (2)					
	OAGB	800 mg OD	8 y	6 mg/L					
Palbociclib capsules (n = 3) High risk	RYGB	125 mg OD	4–5 y	13–34 µg/L (2)	> 61 µg/L	80.3 ± 26.7 µg/L (range 21.2–130 µg/L) [32]	↓	–	Switch to tablets
	SG	125 mg OD	14 y	64–80 µg/L					
	Unknown	125 mg OD	11 y	23 µg/L					
Letrozole (n = 2) Low risk	RYGB	2.5 mg OD	10 m	25.1 µg/L	> 86 µg/L	88.4 µg/L (range 7 [LLOQ]—349.2 µg/L) [33]	↔	–	Start with the usual dose *Consider TDM-guided dosing* < *2 years after surgery*
		5 mg OD	12–15 m	63.3–74.3 µg/L (2)					
		2.5 – 5 mg	21–26 m	99.6–119 µg/L** (2)					
	Unknown	2.5 mg BD	11 y	90.4 µg/L					
		5 mg OD	11 y	239 µg/L					
		2.5 mg OD	11 y	79.4–169 µg/L (2)					
Olaparib (n = 2) Medium risk	SG	250 mg BD	Before surgery (−7–9 m)	765–900 µg/L (2)	Not reported	1514 µg/L (IQR 1170–1740) [34]	?	–	TDM-guided dosing
		200 mg BD	Before surgery (−4 m)	836 µg/L					
	RYGB	300 mg BD	8–9 y	805–1069 µg/L (2)					
Abiraterone (n = 1) Medium risk	RYGB	1000 mg OD	Unknown, after surgery	15–21 µg/L	> 8.4 µg/L	10.9 ± 4.8 µg/L [35]	?	–	TDM-guided dosing
Dabrafenib (n = 1) Medium risk	RYGB	75 mg OD	17 y	56–57 µg/L (2)	> 46.6 µg/L	15.4–279.6 µg/L [36]	?	–	TDM-guided dosing
Imatinib (n = 1) Medium risk	RYGB	300 mg OD	7 y	> 1637–2506 µg/L (3)	> 1100 µg/L	1530 ± 666 µg/L (400 mg OD), 1752 ± 794 µg/L (600 mg OD) [37]	↓	400 mg OD: 629–836 ng/mL (−46 to −60% compared to preoperatively) [38]	TDM-guided dosing
		400 mg OD	7 y	260–1791 µg/L (2)					
		600 mg OD	7 y	750 µg/L					
Sorafenib (n = 1) High risk	RYGB	200 mg OD	31 m	1550 µg/L	Not reported for the indication of desmoid tumor	Not reported for the indication of desmoid tumor	?	–	Switch to a lower-risk drug
		400 mg OD	4 y	300 µg/L					
		400 mg OD 5 days, 2 days off	4 y	< 293 µg/L					
		400 mg OD	5 y	571 µg/L					
Sunitinib (malate) (n = 1)*** Medium risk	RYGB	50 mg OD	11 m	42 mg/L	> 50 mg/L	16–92 mg/L [39]	↓	50 mg OD: 34 mg/L 62.5 mg OD: 37 mg/L 75 mg OD: 53 mg/L [9]	TDM-guided dosing

Total N = 22 patients. Drugs are first sorted by number of patients and then alphabetically. The last column of practical recommendation is based on the flowchart and the available TDM data

Trough concentrations below the therapeutic window are indicated in red. Medians with standard deviations or ranges are provided unless indicated otherwise

* = indicated for OAGB, RYGB, and SG. **asymmetric dosing letrozole. Due to the long elimination half-life of this drug, the measured levels were interpreted as trough levels. ***All measured levels and target concentrations refer to the sum of sunitinib and desethylsunitinib. ^#^Trough concentration based on one sample, unless followed by (n samples). ^##^Target concentrations based on TDM practice in our center

Abbreviations: *BD*  twice daily, *GB* gastric banding, *IQR* interquartile range, *OAGB* one anastomosis gastric bypass, *OD* one time daily, *ref*  reference range, *RYGB* Roux-en-Y gastric bypass, *SG* sleeve gastrectomy

Of the 42 patients treated with tamoxifen, at least one plasma or serum sample was available from nine patients. Of these patients, seven patients had samples drawn after bariatric surgery and six of these patients started with a tamoxifen dose of 20 mg OD. A dose escalation to 40 mg OD was performed in four of these patients due to low endoxifen levels. For two patients, an increased tamoxifen dose led to therapeutic endoxifen levels. From two patients, pre- and post-surgery tamoxifen TDM data were available. The limited follow-up data (i.e., two to six months after surgery) indicated that tamoxifen levels decreased after bariatric surgery.

Of the four pazopanib patients, the samples containing therapeutic pazopanib levels were derived from two patients who underwent GB. The other measured pazopanib trough levels were subtherapeutic and measured in patients who underwent RYGB or OAGB. For one of these patients, the dose of 800 mg OD was changed to 400 mg BD, as described previously [21]. This intervention led to increased plasma levels of pazopanib varying between 13–23 mg/L, barely reaching the target of at least 20 mg/L.

### Flowchart

To evaluate the flowchart that assesses the risk of underdosing due to bariatric surgery (Fig. 1), the classification according to the flowchart for each drug was included in Table 2. Generally, measured levels of the three risk categories were in line with the flowchart, except for letrozole, which was the only drug classified as low risk. Of the two patients who received letrozole, one patient had adequate exposure. Six drugs were classified as medium risk and three of them required TDM-guided interventions. Of 15 patients receiving medium-risk anticancer drugs, 7 (47%) did not reach adequate exposure.

Three drugs were classified as high risk. For palbociclib and pazopanib, no adequate exposure was reached in 4/7 patients (57%), while adequate exposure was reached in two GB patients receiving pazopanib. For sorafenib, the target of exposure was unknown. Unfortunately, for the other high-risk drugs erlotinib, vinorelbine, and selpercatinib (Supplementary Table S1) no TDM data was available. Both patients who received oral vinorelbine in our study did not experience any neutropenia or other side effects.

## Discussion

In this observational study, potential drug-related problems with oral anticancer drugs were investigated in patients who underwent bariatric surgery. In addition, a practical flowchart (Fig. 1) was proposed to categorize the risk of underdosing due to bariatric surgery. This flowchart can help pharmacists and clinicians in the treatment with oral anticancer drugs in patients who underwent bariatric surgery. We evaluated these findings retrospectively with data from a limited set of oral anticancer drugs in SG, RYGB, or OAGB patients. We identified that most patients received an orally administered anticancer drug that was classified as medium or high risk. Unfortunately, no data on drugs with BCS class III were available.

To our knowledge, this is the first study that evaluated the use of various oral anticancer drugs in a cohort of patients who underwent bariatric surgery. We showed that many patients in our comprehensive cancer center (0.4%) had undergone bariatric surgery, which is comparable to the estimated prevalence of bariatric surgery in the Dutch general population (0.6%) [22]. In fact, the number may have been underestimated, as oncologists may not have been aware of all of their patients who had previously undergone bariatric surgery.

The exposure to low-risk oral anticancer drugs was not expected to be influenced by bariatric surgery. This was previously observed in a case report of temozolomide (low risk) in a patient with an RYGB [23]. However, our data showed that a patient who underwent bariatric surgery less than two years ago received letrozole (low risk) the exposure was low. Thus, we cannot rule out that a short period after bariatric surgery potentially influences the exposure to low-risk drugs. Therefore, TDM of BCS class I drugs can be considered if the patient underwent bariatric surgery < 2 years ago.

As for medium-risk oral anticancer drugs, we propose that TDM should be considered to prevent underdosing. As an illustrative example, previous case reports show that bariatric patients treated with tamoxifen (medium-risk) [10] are underdosed with 20 mg OD. These results were confirmed by this study. Therefore, it should be considered to start with a higher dose of tamoxifen.

In contrast, high-risk category drugs should preferably be avoided, as the exposure is likely to be inadequate even with a TDM-guided dosing strategy. These drugs already possess a high risk of underdosing in patients without bariatric surgery, as shown for pazopanib [24]. The likelihood of reaching adequate exposure is even lower in bariatric patients, as previously demonstrated by Tardy et al*.* [25]. In contrast to pazopanib, there is no literature or TDM data available about vinorelbine, another high-risk drug, in patients with bariatric surgery. Two patients in our cohort received vinorelbine [26]. Since vinorelbine has both a low biological availability and a pH-dependent solubility similar to pazopanib, we argue that no adequate exposure to vinorelbine was probably reached in these patients. Besides the general principles as demonstrated in the flowchart, pharmacists and clinicians should also consider sources of inter-individual variability, including therapeutic adherence, drug-drug interactions, and type of bariatric surgery for the final choice of medication or dosing. Other relevant factors related to bariatric surgery or obesity that should also be considered are included in other reviews [27, 28] or guidelines elsewhere [29].

Most patients in our cohort underwent bariatric surgery before their anticancer treatment. Only two patients underwent bariatric surgery after initial treatment with oral anticancer drugs (e.g., antihormonal therapy). In such cases, plasma samples should preferably have been drawn before and after bariatric surgery to assess the effect of the surgery itself. Unfortunately, the follow-up of TDM-guided dosing after bariatric surgery was limited to two patients in our cohort. Both follow-ups were relatively short (i.e., 2–6 months). We recommend clinicians perform TDM more intensively, especially in the first year after bariatric surgery.

Although we believe that this study provides relevant information about oral anticancer drug use in patients who underwent bariatric surgery, it also has several limitations. First, selection bias could have occurred because this study was performed at a comprehensive cancer center. Another limitation of this study is that we included a limited number of patients per anticancer drug. Due to the diversity in the type of cancer, and the different oral anticancer drugs these patients use, it was impossible to draw conclusions about the efficacy or toxicity of the oral anticancer drugs used by patients who underwent bariatric surgery. The type of bariatric surgery could also have had different consequences for the absorption of oral anticancer drugs. Furthermore, it should be acknowledged that in this study missing data (i.e., patient characteristics, prescriptions, and plasma levels) was introduced due to the presence of second opinions and referrals. However, we argue that these missing data only lead to an underestimation of possible drug-related problems in patients who underwent bariatric surgery receiving oral anticancer drugs. Lastly, we did not include supportive care (i.e., oral premedication, corticosteroids), while drug-related problems with these important drugs may be present.

In this retrospective analysis, we identified several important signals that warrant action. First, it is crucial to accurately record bariatric surgery in the medical files of cancer patients. Second, this information should be taken into account before the final choice is made for the anticancer drug and dose, where our flowchart may be helpful. Third, further research is recommended to investigate the effect of bariatric surgery on the pharmacokinetics of orally administered anticancer drugs. Subsequently, this information should become widely available in clinical decision support systems, as is the case for example for reduced renal function. Until this information has become available, we argue that increased awareness is needed in the treatment with orally administered anticancer drugs in patients who underwent bariatric surgery. This topic is considered increasingly relevant due to the growing number of patients who underwent bariatric surgery. Bariatric patients with oral anticancer drugs deserve additional monitoring and may need to switch to other drugs with a lower risk of underdosing.

## Conclusion

Dosing of most oral anticancer drugs can be challenging in patients who underwent bariatric surgery. Generally, lower plasma levels may be anticipated, putting bariatric patients at risk of being underdosed. Based on the drug properties, we recommend considering TDM in drugs that belong to BCS classes II, III, or IV. If more risk factors are present in BCS classes II or IV, a priori switches to other drugs may be advised. In specific cases, higher dosages can be provided from the start (e.g., tamoxifen).

### Supplementary Information

Below is the link to the electronic supplementary material.

Appendix 1 (DOCX 15 KB)

Supplementary Table S1 (DOCX 18 KB)

Supplementary Table S2 (DOCX 23 KB)

## Data Availability

The data that support the findings of this study are available from the corresponding author on reasonable request. The data are not publicly available due to privacy or ethical restrictions.
