# Supplementary material for: Impact of bariatric surgery on oral anticancer drugs: an analysis of real-world data
Source: Cancer Chemother Pharmacol. 2024 Mar 1;94(1):25–34. doi: 10.1007/s00280-024-04640-0 (PMC11258081; doi:10.1007/s00280-024-04640-0)
Supplement: Supplementary file 1 — Appendix 1 (DOCX 15 KB) [file 280_2024_4640_MOESM1_ESM.docx]

## **Appendix 1 Used search terms for extraction of patients with bariatric surgery**

These equivalent Dutch search terms were used to identify patients with bariatric surgery in their medical history:

- “Bariatric” OR “bariatrics”
- “Weight loss surgery” OR “Weight reduction surgery”
- “Gastric bypass” OR “RYGB”
- “Gastric banding”
- “Gastric sleeve” OR “Sleeve gastrectomy” OR “SG”
- “Duodenal switch”
- “Biliopancreatic diversion” OR “BPD”
- “One anastomosis gastric bypass” OR “OAGB”
- “Single anastomosis stomach ileal bypass” OR “SASI”
- “Single anastomosis duodeno ileal bypass” OR “SADI”
