# Supplementary material for: Impact of bariatric surgery on oral anticancer drugs: an analysis of real-world data
Source: Cancer Chemother Pharmacol. 2024 Mar 1;94(1):25–34. doi: 10.1007/s00280-024-04640-0 (PMC11258081; doi:10.1007/s00280-024-04640-0)
Supplement: Supplementary file 2 — Supplementary Table S1 (DOCX 18 KB) [file 280_2024_4640_MOESM2_ESM.docx]

**Table S1. Overview of oral anticancer drugs administered to the included patients and the corresponding BCS class, formulation, and whether the absorption is dependent on gastric pH.** Total N of 152 patients. Drugs are sorted by number of patients and then alphabetically

| **Anticancer drug** | **Patients (n)** | **BCS class** [14, 15] | **Influence of gastric pH** | **Biological availability (%)*** | **Enabling formulation** [14] | **Class based on flowchart**** |
| --- | --- | --- | --- | --- | --- | --- |
| Tamoxifen | 42 | II | No | 100 | No | Medium risk |
| Letrozole | 25 | I | No | 100 | No | Low risk |
| Capecitabine | 17 | I | No | 100 | No | Low risk |
| Anastrozole | 13 | II | No | UNK | No | Medium risk |
| Bicalutamide | 9 | II | No | UNK | Lipid-based capsule | Medium risk |
| Exemestane | 8 | II / IV | No | UNK | No | Medium risk |
| Pazopanib | 4 | II | Yes | 21 [40] | No | High risk |
| Temozolomide | 4 | I | No | 100 | No | Low risk |
| Palbociclib | 3 | IV | Yes (capsules) | 46 [41] | No | High risk |
|  | 0 |  | No (tablets) |  |  | Medium risk |
| Cyclophosphamide | 2 | I | No | >85 | No | Low risk |
| Erlotinib | 2 | II | Yes | 59 | No | High risk |
| Lomustine | 2 | II / IV | No | ≥73 | No | Medium risk |
| Procarbazine | 2 | UNK | No | ≥70 | No | Medium risk |
| Ribociclib | 2 | II | No | 65.8 [42] | No | Medium risk |
| Sorafenib | 2 | II | Yes | UNK | No | High risk |
| Vinorelbine | 2 | IV | Yes | 36 | Co-solvent | High risk |
| Abiraterone acetate | 1 | IV | No | ≤10 | No | Medium risk |
| Binimetinib | 1 | I (acidic pH) /  II (physiological pH) | No | UNK | No | Medium risk |
| Cabozantinib | 1 | II | No | UNK | No | Medium risk |
| Chlorambucil | 1 | UNK | No | >70 | No | Medium risk |
| Dabrafenib | 1 | II | No | 95 | No | Medium risk |
| Encorafenib | 1 | II | No | UNK | No | Medium risk |
| Enzalutamide | 1 | II | No | ≥84 | Lipid-based capsule | Medium risk |
| Everolimus | 1 | IV | No | UNK | Solid dipersion | Medium risk |
| Imatinib | 1 | II | No | 98 | No | Medium risk |
| Olaparib | 1 | IV | No | UNK | No | Medium risk |
| Selpercatinib | 1 | IV | Yes | 73 [43] | No | High risk |
| Sunitinib (malate) | 1 | IV | No | UNK | No | Medium risk |
| Sunitinib (base) | 0 | IV | Yes | UNK | No | High risk |
| Trametinib | 1 | IV | No | 72 | No | Medium risk |

* Biological availability extracted from reference [14], unless indicated otherwise. ** Assuming SG < 2 years, RYGB and OAGB

Abbreviations: BCS = biopharmaceutical classification system, UNK = unknown
