# Supplementary material for: Impact of bariatric surgery on oral anticancer drugs: an analysis of real-world data
Source: Cancer Chemother Pharmacol. 2024 Mar 1;94(1):25–34. doi: 10.1007/s00280-024-04640-0 (PMC11258081; doi:10.1007/s00280-024-04640-0)
Supplement: Supplementary file 3 — Supplementary Table S2 (DOCX 23 KB) [file 280_2024_4640_MOESM3_ESM.docx]

**Table S2. Overview of measured serum or plasma levels and used drug doses in patients before or after GB.** Total N=3 patients. Drugs are first sorted by number of patients and then alphabetically.

| **Drug (n patients)**  **Risk based on flowchart** | **Type of surgery** | **Dosage** | **Time after surgery** | **Trough concentration^#^** | **Target concentra-tion^##^** | **Expected concentration range in non-bariatric patients** | **Expected trend in bariatric patients with GB** | **Practical recommendation** |
| --- | --- | --- | --- | --- | --- | --- | --- | --- |
| Pazopanib (n=2)  High risk | GB | 800 mg OD | 34 y | 27 mg/L | > 20 mg/L | 800 mg OD:  29.7 mg/L (range 0.0 - 83.2 mg/L) [31] | ↔ | As clinical practice |
|  | GB | 800 mg OD | 6 y | 27 mg/L |  |  |  |  |
|  |  |  | 7 y | 37 mg/L |  |  |  |  |
| Tamoxifen (n=1)  Medium risk | GB | 20 mg OD | Unknown, after surgery | 18.4 ng/mL | > 5.9 ng/mL | 0-73.7 ng/mL (IQR 4.2 - 26.2 ng/mL) [30] | ↔ | As clinical practice |

Trough concentrations below the therapeutic window are indicated in red. Medians with standard deviations or ranges are provided unless indicated otherwise

^#^Trough concentration based on one sample, unless followed by (n samples) ^##^ Target concentrations based on TDM practice in our center.

Abbreviations: GB = gastric banding, IQR = interquartile range, OD = one time daily, ref = reference range
